# Functions of Aurora kinase C in meiosis and cancer

**DOI:** 10.3389/fcell.2015.00050

**Published:** 2015-08-20

**Authors:** Suzanne M. Quartuccio, Karen Schindler

**Affiliations:** Department of Genetics, Rutgers, The State University of New JerseyPiscataway, NJ, USA

**Keywords:** meiosis, Aurora kinase, cancer, fertility

## Abstract

The mammalian genome encodes three Aurora kinase protein family members: A, B, and C. While Aurora kinase A (AURKA) and B (AURKB) are found in cells throughout the body, significant protein levels of Aurora kinase C (AURKC) are limited to cells that undergo meiosis (sperm and oocyte). Despite its discovery nearly 20 years ago, we know little about the function of AURKC compared to that of the other 2 Aurora kinases. This lack of understanding can be attributed to the high sequence homology between AURKB and AURKC preventing the use of standard approaches to understand non-overlapping and meiosis I (MI)-specific functions of the two kinases. Recent evidence has revealed distinct functions of AURKC in meiosis and may aid in our understanding of why chromosome segregation during MI often goes awry in oocytes. Many cancers aberrantly express AURKC, but because we do not fully understand AURKC function in its normal cellular context, it is difficult to predict the biological significance of this expression on the disease. Here, we consolidate and update what is known about AURKC signaling in meiotic cells to better understand why it has oncogenic potential.

## Discovery and genomic features

Three laboratories independently discovered *AURKC* and reported high transcript levels in testes and oocytes (Gopalan et al., 1997; Bernard et al., 1998; Tseng et al., 1998). A subsequent study reported low expression of *AURKC* in some normal somatic cells including skeletal muscle, placenta, lung and bladder (Yan et al., 2005b) although germ cell expression is much higher (49 times) (Assou et al., 2006). In addition, Kimura et al. (1999) found elevated levels of AURKC in breast, cervical, and liver cancer cells lines.

*AURKC* is a member of the conserved serine/threonine Aurora kinase family. These kinases are related to *Increase-in-ploidy1* in budding yeast and *Aurora* in *Drosophila*, both of which regulate spindle formation and chromosome segregation (Francisco and Chan, 1994; Glover et al., 1995). Yeast contains one Aurora kinase (Petersen et al., 2001), while *Drosophila, C. elegans* and *Xenopus* express two (Roghi et al., 1998) generated from gene duplication in cold-blooded vertebrates (Brown et al., 2004). The mammalian genome encodes three Aurora kinases. *AURKC* is located on human Chromosome 19 [19q13.43 (Kimura et al., 1999)] and *Aurkc* on mouse Chromosome 7 A2-A3 (Gopalan et al., 1997). Human AURKC shares 82.1 and 68.8% amino acid identity with mouse AURKC in the kinase and N-terminal domains, respectively however only 26.7% identity in the C-terminal domain suggesting species-specific differences (Tseng et al., 1998).

**Figure 1:**
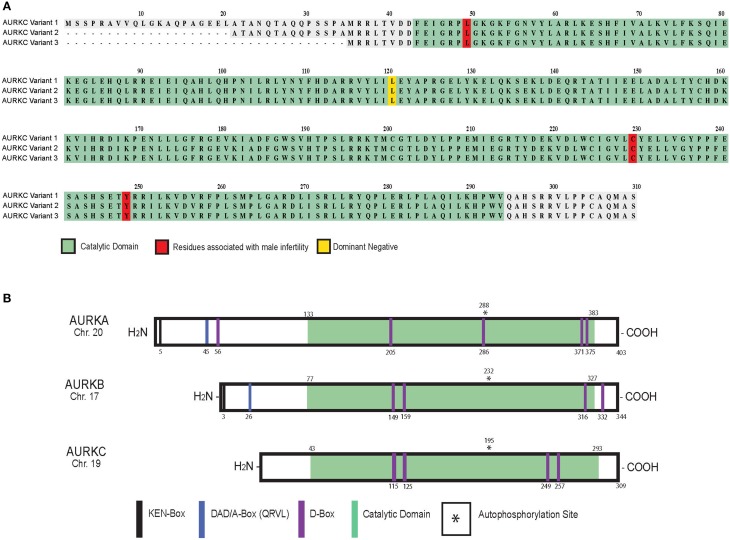
**AURKC variants and Aurora kinase family members in mammals**. Schematic of human AURKC variants **(A)** and Aurora kinase isoforms **(B)** with key domains and residues identified.

Alternative splicing results in three protein variants of AURKC (Bernard et al., 1998; Tseng et al., 1998; Yan et al., 2005b) (Figure 1A). Variants 2 and 3 lack amino acid residues in the N-terminus that do not appear to regulate localization (Fellmeth et al., 2015). While all variants are catalytically active, variant 1 is better at phosphorylating targets in oocytes suggesting the N-terminus positively regulates activity. Human oocytes contain all three variants while only one or two variants are measured in sperm (Fellmeth et al., 2015).

At the protein level, AURKC shares sequence homology with AURKA (60% identical) and AURKB (75% identical) in the kinase domain (Quintas-Cardama et al., 2007). Autophosphorylation of a threonine contained within the activation loop (T-loop) activates the kinases (Figure 1B) (Goldenson and Crispino, 2015). AURKC lacks the N-terminal domain found in AURKA and B (Gopalan et al., 1997; Kimura et al., 1999) containing the KEN (KENXXX) and D-box activating domain (DAD/A-box, QRVL) motifs suggesting that it is differentially regulated. The anaphase promoting complex/cyclosome (APC/C) recognizes these sequences and marks the protein for degradation (Nguyen et al., 2005). AURKB and AURKC do contain four D-box motifs (RXXL), which can be recognized by the APC/C, however their regulatory function is unknown (Nguyen et al., 2005; Stewart and Fang, 2005; Schindler et al., 2012).

## AURKC signaling in sperm

### Spatiotemporal regulation

Localization of AURKC in spermatocytes is dynamic and linked to its function. Mouse spermatocytes express measurable levels of AURKC protein at centromeres in the diplotene stage of prophase (Tang et al., 2006) followed by localization at centromeres and along chromosome arms during diakinesis (Tang et al., 2006). Next, AURKC translocates to the spindle midzone at anaphase I and the midbody at telophase I. AURKC follows the same distribution pattern through meiosis II (MII) (Tang et al., 2006) then dissociates from centromeres (Tang et al., 2006). Human spermatocytes exhibit the same localization pattern of AURKC (Avo Santos et al., 2011). AURKC co-localizes with AURKB and immunoprecipitates with INCENP in spermatocytes suggesting that it is a member of the meiotic chromosomal passenger complex (CPC) (Tang et al., 2006) that regulates chromosome alignment and condensation, kinetochore-microtubule attachments (K-MT) and cytokinesis (Sharif et al., 2010; Yang et al., 2010; Balboula and Schindler, 2014).

### Expression levels

*Aurkc* expression is also regulated in a stage-specific manner (Kimmins et al., 2007). *In situ* hybridization revealed positive expression in some seminiferous tubules from mice with meiotic cells in prophase (4C) having the highest levels (Tang et al., 2006). *Aurkc* transcript first appears in the testes of mice 14 days after birth (Hu et al., 2000). mRNA levels increase and plateau at day 21 before decreasing at day 28, but mRNA is still observed at day 42 (Hu et al., 2000).

### Male fertility

Male *Aurkc*^−/−^ mice are viable with normal testis weight and sperm counts but are subfertile (Kimmins et al., 2007). This subfertility is attributed to blunted sperm heads, defects in chromatin condensation and acrosome detachment (Kimmins et al., 2007). In humans, AURKC is essential for male fertility. Current studies indicate that mutations in AURKC are the most frequent genetic cause of macrozoospermia (Ounis et al., 2015), a condition where ~100% of a patient's sperm have large, misshapen heads. These sperm have multiple flagella (Dieterich et al., 2009) due to a meiotic arrest in MI (Dieterich et al., 2007) suggesting AURKC is critical for cytokinesis.

A genome-wide microsatellite scan of 10 affected men from the Rabat region of Morocco revealed cysteine deletion in exon 3 of *AURKC* (c.144delC, also called L49W) (Figure 1A). The mutation induces a frameshift leading to premature termination of translation and truncated protein (Dieterich et al., 2007). A subsequent study found that the mutation, which also induces non-sense mediated mRNA decay (Ben Khelifa et al., 2011), occurs at a rate of 1 in 50 in the Maghrebian population (Dieterich et al., 2009) suggesting a selective advantage for harboring this allele. Heterozygous mutations of c.144delC combined with C229Y, Y248X (Dieterich et al., 2009) or c.436-2A>G required for proper slicing (Ben Khelifa et al., 2011) produced a similar phenotype. Few morphologically “normal” sperm can be isolated from these men and used for intracytoplasmic injection into eggs. However, euploid embryos were never generated indicating that sperm from men with AURKC mutations cannot be used in the *in vitro* fertilization clinic (Ben Khelifa et al., 2011; El Kerch et al., 2011).

Interestingly, women homozygous for c.144delC are not sterile indicating a sexually dimorphic role of AURKC. But the small sample size (*n* = 2) of the study limits the impact of this finding (Dieterich et al., 2009).

## AURKC signaling in oocytes

### Spatiotemporal regulation

Mammalian oocytes display dynamic localization of AURKC. AURKC localizes to centromeres and along chromosome arms during prometaphase and metaphase I before concentrating at the midzone and midbody during anaphase I and telophase I, respectively (Uzbekova et al., 2008; Avo Santos et al., 2011). AURKC's localization at the interchromatid axis (ICA) of bivalents at metaphase of MI is regulated by haspin in mouse oocytes (Nguyen et al., 2014) and distinguishes the kinase from AURKB that is found on the spindle. Therefore AURKC localization in sperm and oocytes is identical.

### Expression levels

In oocytes, *Aurkc* expression is also regulated temporally. The relative mRNA level of *Aurkc* in prophase I-arrested mouse oocytes is similar compared to mRNA levels of *Aurkb* but 9–20 fold less than *Aurka* (Shuda et al., 2009; Schindler et al., 2012). Oocytes that are competent to complete meiosis are transcriptionally silent. This silence persists until zygotic genome activation. To ensure plentiful protein stores, oocytes recruit maternal messages for translation during MI through a cytoplasmic polyadenylation element in the 3′ untranslated region of genes. *Aurkc* contains this element and is recruited (Schindler et al., 2012). Therefore although *Aurkc* mRNA levels drop to undetectable levels in blastocysts, (Avo Santos et al., 2011; Schindler et al., 2012) protein remains in the embryo until AURKB becomes the predominant CPC kinase (Fernandez-Miranda et al., 2011; Schindler et al., 2012).

### Female fertility

Female *Aurkc*^−/−^ mice survive but are subfertile due to meiotic abnormalities and compromised embryonic development (Schindler et al., 2012). Oocytes from *Aurkc*^−/−^ mice often contain misaligned chromosomes and arrest at MI. Some oocytes do undergo cytokinesis and extrude a polar body but are delayed. In addition, fewer one-cell embryos from *Aurkc*^−/−^ mice reach the two-cell stage due to cytokinesis failure, and this phenotype worsens during development (Schindler et al., 2012).

While overexpression of AURKB can rescue MI arrest and cytokinesis failure (Schindler et al., 2012) endogenous levels of AURKC are sufficient for preimplantation embryonic development *Aurkb*^−/−^ embryos (Fernandez-Miranda et al., 2011). These phenotypic data combined with the instability of AURKB and recruitment of *Aurkc* messages during MI (Schindler et al., 2012) drove the conclusions that mouse oocytes require AURKC because AURKB levels are insufficient to ensure completion of meiosis and embryonic mitoses. Importantly, wild-type mouse oocytes expressing a dominant-negative allele of AURKC that does not inhibit AURKB (AURKC-LA) (L93A in mouse [variant 2]; L120A in human [variant 1]) are aneuploid (Balboula and Schindler, 2014). These data indicate that when a non-functional AURKC protein is bound in the CPC, AURKB cannot compete for binding to support meiosis. We anticipate that as more genomes are sequenced, mutations in AURKC that alter activity in the CPC will be correlated with female infertility.

## Overlapping AURKB and AURKC function

The CPC regulates the spindle assembly checkpoint (SAC), cytokinesis and correction of K-MT attachments. AURKC specific inhibition (AURKC-LA) does not alter the localization of SAC component BUB1 in oocytes suggesting that both AURKB and AURKC regulate SAC activation in meiosis (Balboula and Schindler, 2014). Only after microinjection of the dominant negative form of *Aurkc* (*Aurkc-DN*) (T171A/T175A, variant 2), which disrupts the function of both AURKs, is BUB1 localization altered and SAC non-functional (Yang et al., 2010; Balboula and Schindler, 2014). AURKB and C also share regulation of cytokinesis. AURKC-LA-expressing oocytes that complete MI extrude a polar body, while AURKC-DN oocytes retract polar bodies (Kimura et al., 1999; Balboula and Schindler, 2014). In contrast, similar levels of incorrect K-MT attachments (Balboula and Schindler, 2014) are observed in AURKC-LA and AURKC-DN oocytes suggesting AURKC, particularly at the ICA (Nguyen et al., 2014), is the primary CPC kinase to correct attachments (Balboula and Schindler, 2014). In mitotic cells the CPC preferentially binds AURKB (Sasai et al., 2004), but increased translation of AURKC during MI is consistent with AURKC being the preferred catalytic component of the CPC in oocytes (Assou et al., 2006).

AURKB and AURKC share a consensus phosphorylation motif (R-X-S/T-Φ, Φ represents any hydrophobic residue except P)(Alexander et al., 2011) and therefore phosphorylate many of the same substrates. These kinases can bind the “IN box” of INCENP (Tang et al., 2006; Ben Khelifa et al., 2011) leading to autophosphorylation and kinase activation (Li et al., 2004). AURKC binds the other CPC components (Survivin and Borealin) when overexpressed in mitotic cells (Sasai et al., 2004; Chen et al., 2005; Yan et al., 2005a; Slattery et al., 2008, 2009) and phosphorylates histone H3 at S10 in meiotic and mitotic cells (Li et al., 2004; Avo Santos et al., 2011), which may play a role in chromosome condensation (Swain et al., 2008). In addition both AURKB and AURKC phosphorylate Centromere protein A in mitotic cells (Sasai et al., 2004; Slattery et al., 2008), which is required for the recruitment of kinetochore proteins, chromosome segregation and cell cycle progression. Future investigations need to evaluate whether other known downstream targets of AURKB, such as Hec1 (Zhu et al., 2013) and the MAPK pathway (Xu et al., 2012), are also targeted by AURKC.

## Unique AURKC functions

Although AURKB and AURKC often exhibit conserved function, they cannot fully compensate for the loss of one another (Kimmins et al., 2007; Fernandez-Miranda et al., 2011; Schindler et al., 2012; Balboula and Schindler, 2014) indicating that non-overlapping roles exist. Evidence of these differences can be seen in the divergent phenotypes of knockout mice. *Aurkb*^−/−^ die at the blastocyst embryonic stage while *Aurkc*^−/−^ knockouts are viable (Kimmins et al., 2007; Schindler et al., 2012). Transgenic mice expressing a dominant negative AURKB driven by the male-specific β-4-galactosyltransferase promoter exhibit severe disruption in spermatogenesis with reduced sperm counts, reduced testis size and disorganized spermatogenic staging. 48% of these mice are sterile and cytokinesis failure is observed (Kimmins et al., 2007). This inability of AURKC to physiologically compensate for AURKB absence in sperm suggests that AURKB has specific functions in mouse spermatogenesis (Kimmins et al., 2007) although the dominant negative allele used may also affect the function of AURKC.

The non-overlapping functions of AURKB and C have also been demonstrated in experiments with oocytes, consistent with their spatial separation (Balboula and Schindler, 2014). Overexpression of AURKC causes arrest in MI due to cytokinesis failure (Sharif et al., 2010). Securin levels decrease (a sign of APC/C activation) and activated separase triggers homologous chromosome separation (Sharif et al., 2010). This phenotype differs from AURKB-overexpressing oocytes, which fail to activate the APC/C and stabilize securin and have unresolved chiasmata (Sharif et al., 2010). These data indicate that AURKC plays a role in cell cycle progression while AURKB acts to maintain the SAC. Another indication of unique activity: overexpression of AURKB, and not AURKC, can rescue the misaligned and slowed progression phenotype of ZM447439-treated oocytes (Shuda et al., 2009). These data suggest that high levels of AURKB can displace AURKC from the CPC, that AURKB has a non-CPC function, or that AURKB-CPC has a chromosome-independent function. Future studies are critical to decipher other AURKC specific functions.

## Expression of meiotic genes in cancer

Meiomitosis is the expression of meiosis-specific proteins in mitotic cells (Grichnik, 2008) and can negatively impact genetic stability. Meiotic proteins, or cancer testis antigens (CTA), are used as diagnostic and prognostic indicators (Fratta et al., 2011; Rosa et al., 2012) in skin, bladder, lung and ovarian tumors. Upregulation of CPC components, including AURKC, occurs in cancer cells (Yan et al., 2005a) and may correlate with clinical characteristics in primary colorectal cancers (Takahashi et al., 2000; Lin et al., 2014). It is unclear if CTA expression is the initiating oncogenic event or a downstream consequence of transformation (Rosa et al., 2012), but could indicate that cancer cells use meiotic divisions (i.e. separating homologs) for growth and survival advantages (Ianzini et al., 2009). These proteins represent desirable diagnostic biomarkers for tumor subtype and ideal candidates for targeted therapeutics because their expression is limited to germ cells, thereby minimizing side effects.

## AURKC signaling in cancer cells

**Figure 2:**
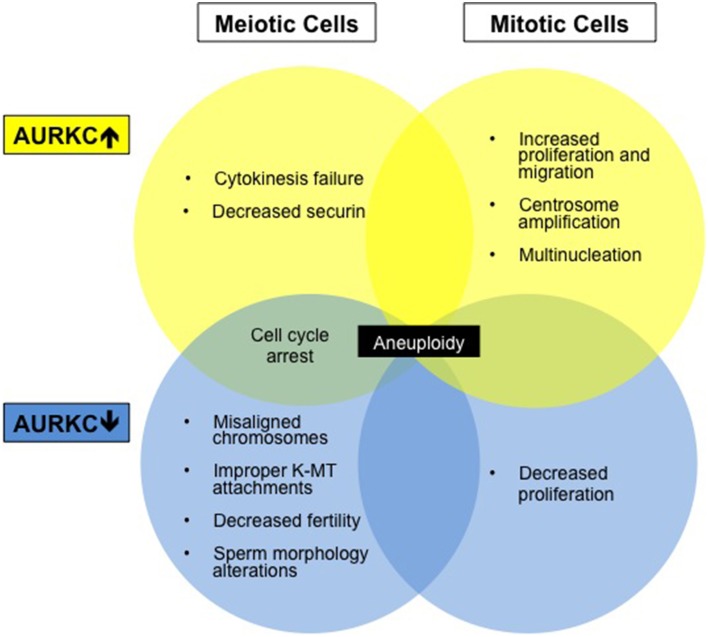
**Aberrations in AURKC levels results in altered cell phenotypes**. Diagram summarizing cell phenotypes observed when AURKC expression is disrupted in mitotic and meiotic cells.

*AURKC* is oncogenic because its overexpression transforms NIH 3T3 cells into tumors (Khan et al., 2011). AURKC is overexpressed in many cancer cell lines, including NB1RGB, MDA-MB-453, HEPG2, HeLa, and HuH7 (Kimura et al., 1999), and in cancer of the reproductive tract (Tsou et al., 2011). Overexpression increases cellular proliferation and migration and enhances xenograft tumor growth (Tsou et al., 2011). Kinase-dead AURKC decreases proliferation of HeLa cells while expression of the constitutively active AURKC (Spengler, 2007a; Khan et al., 2012) leads to more aggressive tumors (Khan et al., 2011; Tsou et al., 2011). Other carcinogenic genes are also located in the telomeric region of human Chromosome 19 (Bernard et al., 1998), a genomic region susceptible to translocations and deletions (Bernard et al., 1998; Kimura et al., 1999). Although some plasticity exists between the Aurora kinase family members allowing for functional compensation; some roles are kinase specific and maintaining the correct balance is necessary for genomic stability (Figure 2).

The functional significance of AURKC expression in cancer cells is unknown but may relate to centrosome regulation. Overexpression of AURKC in mitotic cells leads to centrosome amplification and multinucleation (Khan et al., 2011), a hallmark of cancer. Extra centrosomes are associated with the formation of multipolar spindles. Multipolar spindle formation usually leads to cell death however centrosome clustering appears to support cancer cell survival and frequently leads to chromosome segregation defects (Marthiens et al., 2012). AURKC localizes to centrosomes with AURKA during interphase (Takahashi et al., 2000; Dutertre et al., 2005) and may play a role in centrosome clustering. Many new cancer therapies are aimed at declustering centrosomes (Pannu et al., 2014) which forces cancer cells to form a multipolar spindle and induces cell death. AURKC inhibition may alter this clustering pathway.

AURKC interactions with other proteins linked to cancer may also explain its oncogenic role. AURKC, as well as AURKA and AURKB, phosphorylate the transforming acidic coiled-coil 1 protein (TACC1) (Gabillard et al., 2011). Overexpression of *TACC1* drives cell transformation (Cully et al., 2005) and serves as a prognostic marker of endocrine therapy resistance in breast cancer (Ghayad et al., 2009). AURKC also phosphorylates TRF2, a protein involved in telomere length regulation (Spengler, 2007b). Decreased telomere length predisposes individuals to cancer (Shammas, 2011) and negatively impacts fertility (Spengler, 2007b). In addition, tumor necrosis factor alpha induces increased AURKC expression through the inflammation response factor CEBPD in HeLa cells (Wu et al., 2011). Ongoing studies of normal AURKC functions in meiotic cells are critical to improving our understanding of the role of aberrant expression in cancer.

## Small molecule inhibitors

More than 70 clinical trials have been conducted on Aurora kinase inhibitors. First generation inhibitors failed due to low efficacy and high toxicity (Goldenson and Crispino, 2015) however second-generation inhibitors are more sub-type specific which may alleviate side effects. SNS-314 is a pan-Aurora kinase inhibitor (Oslob et al., 2008) with AURKA, B and C IC50 values of 9, 31, and 3 nM, respectively (Kollareddy et al., 2012). This ATP-competitive inhibitor can inhibit proliferation of anaplastic thyroid cancer cells *in vitro* (Baldini et al., 2012) and inhibit tumor growth of colon cancer xenografts (Arbitrario et al., 2010). A phase I clinical trial on advanced solid tumors showed modest results of SNS-314 treatment alone (Robert et al., 2008), however sequential administration of SNS-314 and chemotherapy docetaxel exhibited synergistic anti-proliferative effects (VanderPorten et al., 2009). AMG-900 inhibits AURKC with a 1nM IC50 (Payton et al., 2010). The compound induces apoptosis in a diverse set of cancer cell lines *in vitro* and inhibits tumor growth *in vivo* (Payton et al., 2010) AMG-900 inhibits colony formation of multidrug resistant cell lines (Payton et al., 2010; Bush et al., 2013) and shows additive effects when combined with histone deacetylase inhibitors (Paller et al., 2014). Two Phase I clinical trials are being conducted on advanced solid tumors and acute leukemias (Kollareddy et al., 2012).

## Conclusion

Many advances have been made regarding our knowledge of AURKC as a regulator of chromosome segregation, but many questions remain. Does AURKC have unique, MI-specific substrates and do they differ between sperm and oocyte? What cofactors are needed for full AURKC activation? Does AURKC function outside of the CPC? Does AURKC drive meiotic events when expressed in mitotic cells giving rise to tumors? Not until we have a complete understanding of the function and substrates of AURKC in meiotic cells can we begin to understand the significance of its expression in cancer cells. However, once these and other meiomitotic protein studies are complete, this class of proteins represent a promising diagnostic and therapeutic cancer target.

## Funding

SQ was supported by an NIH grant K12-GM093854. This work is supported by a NIH grants R01-GM112801 and P30 CA072720 to KS.

### Conflict of interest statement

The authors declare that the research was conducted in the absence of any commercial or financial relationships that could be construed as a potential conflict of interest.

## Abbreviations

APC/C: Anaphase promoting complex/cyclosome
AURKA: Aurora kinase A
AURKB: Aurora kinase B
CPC: Chromosomal passenger complex
ICA: Interchromatid axis
K-MT: Kinetochore microtubule
MI: Meiosis I
MII: Meiosis II
SAC: Spindle assembly checkpoint
TACC1: Transforming acidic coiled-coil 1.
